# Anti-inflammatory activity of palmitoylethanolamide ameliorates osteoarthritis induced by monosodium iodoacetate in Sprague–Dawley rats

**DOI:** 10.1007/s10787-021-00870-3

**Published:** 2021-09-01

**Authors:** Jae In Jung, Hyun Sook Lee, Young Eun Jeon, So Mi Kim, Su Hee Hong, Joo Myung Moon, Cho Young Lim, Yoon Hee Kim, Eun Ji Kim

**Affiliations:** 1 Regional Strategic Industry Innovation Center, Hallym University, 1 Hallymdaehak-gil, Chuncheon, Gangwon 24252 Republic of Korea; 2 Department of Food Science and Nutrition, Dongseo University, Busan, 47011 Republic of Korea; 3 Technology Development Center, BTC Corporation, Ansan, Gyeonggi 15588 Republic of Korea

**Keywords:** Osteoarthritis, Palmitoylethanolamide (PEA), Inflammation, Cytokine, Monosodium iodoacetate (MIA)

## Abstract

Novel treatment strategies are urgently required for osteoarthritis (OA). Palmitoylethanolamide (PEA) is a naturally occurring fatty acid amide with analgesic and anti-inflammatory effects. We aimed to examine its effect on OA and elucidate the molecular mechanism of actions in monosodium iodoacetate (MIA)-induced OA Sprague–Dawley rats. The experimental animals were divided into normal control group (injected with saline + treated with phosphate-buffered saline (PBS), NOR), control group (injected with MIA + treated with PBS, CON), 50 or 100 mg/kg body weight (BW)/day PEA-treated group (injected with MIA + treated with 50 or 100 mg of PEA/kg BW/day, PEA50 or PEA100), and positive control group (injected with MIA + treated with 6 mg of diclofenac/kg BW/day, DiC). The changes in blood parameters, body parameters, gene expression of inflammatory mediators and cytokines, knee thickness, and joint tissue were observed. Oral administration of PEA had no adverse effects on the BW, liver, or kidneys. PEA reduced knee joint swelling and cartilage degradation in MIA-induced OA rats. The serum levels of leukotriene B4, nitric oxide, tumor necrosis factor (TNF)-α, interleukin (IL)-1β, and prostaglandin E2 considerably reduced in the PEA100 group compared with those in the CON group. In the synovia of knee joints, the mRNA expression of *iNOS*, *5-Lox*, *Cox-2*, *Il-1β*, *Tnf-α*, and *Mmp-2*, *-3*, *-9*, and *-13* apparently increased with MIA administration. Meanwhile, *Timp-1* mRNA expression apparently decreased in the CON group but increased to the normal level with PEA treatment. Thus, PEA can be an effective therapeutic agent for OA.

## Introduction

Osteoarthritis (OA) is a common progressive and degenerative joint disease that arises from joint cartilage destruction and joint tissue repair induced by inflammatory mediators (Alshami 2014; Steels et al. 2019). OA results from the failure of chondrocytes to control the balance between the synthesis and degradation of extracellular matrix components in articular cartilage (Man and Mologhianu 2014). The symptoms of OA include subchondral bone thickening, synovial inflammation, osteophyte formation, joint capsule hypertrophy, and ligament degeneration (Chen et al. 2017). These symptoms eventually lead to pain, stiffness, and disability, as well as lower the quality of life of the patients (Zhang et al. 2016).

As the cause of OA is unclear, its management is limited to medications to relieve pain, exercise therapy, weight control, prevention of complications such as muscle degeneration and joint deformity, and maintenance of the range of motion of the joint (Mora et al. 2018). Recently, it was reported that neuroinflammation caused by an imbalance between pro-inflammatory and pro-resolving mediators plays an important role in pain progression (Paladini et al. 2016). Chronic decline in immunity and increase in oxidative stress due to a natural immune response is observed in patients with OA (Chen et al. 2017; Mora et al. 2018). However, arthralgia is not always proportional to the degree of joint damage and immunity (Hochman et al. 2010). Long-term medications can alleviate OA pain but not cure OA (Bjordal et al. 2007).

Currently, the most commonly used OA pain relievers are non-steroidal anti-inflammatory drugs (NSAIDs), which are not very effective in relieving OA pain and exert adverse effects such as bleeding and gastric ulceration when used for a long period (Hinz and Brune 2004). Therefore, it is necessary to identify more effective and safe treatment strategies (Britti et al. 2017).

Palmitoylethanolamide (PEA), a type of N-acylethanolamine (NAE), belongs to a family of endogenous bioactive lipids and is known to be effective against pain and inflammation (Re et al. 2007). PEA is a potential nutraceutical and a naturally occurring fat-derived signaling compound in many animals and plant foods, as well as in animal cells and tissues. The main food sources of PEA are soy lecithin, soybean, peanut, and corn (Petrosino and Di Marzo 2017). Generally, PEAs are manufactured by reacting palmitic acid and ethanolamine (LoVerme et al. 2005). Ethanolamine is usually derived from fossil fuels, but can also be manufactured through the decarboxylation reaction of serine (Scott et al. 2007). The decarboxylation reaction can be performed using pyridoxal 5'-phosphate-dependent serine decarboxylase, and this enzyme can be isolated from various plants such as spinach (Rontein et al. 2001). Among NAEs, PEA has been the most studied modulator of pain and inflammation (Steels et al. 2019). It has been shown to exert analgesic and anti-inflammatory effects in cells during inflammatory reactions and neurodegenerative conditions (Darmani et al. 2005). It has been reported that PEA increases in the spinal cord of animals with experimentally induced OA, and the PEA concentration decreases in the synovial fluid of patients with OA (Sagar et al. 2010; Richardson et al. 2008). In other words, PEA metabolism occurs abnormally in these diseases, and it is speculated that PEA supplementation may be helpful in treating these diseases or reducing their symptoms. The analgesic effect of PEA has also been reported in two cohort studies of patients with diseases such as OA (Gatti et al. 2012; Hesselink and Hekker 2012). In addition, in a recent study, PEA alleviated pain in patients with knee OA (Steels et al. 2019). However, the effect of PEA on joint disease-related pain is not fully understood, and more studies are needed to confirm the molecular mechanism of PEA action (Britti et al. 2017).

Monosodium iodoacetate (MIA)-induced OA Sprague–Dawley rats have been used as an OA experimental model. To experimentally induce OA in animals, carrageenan has been injected to cause acute and severe inflammation and pain. However, it has been reported that intra-articular injection of MIA is more likely to induce OA and the resulting inflammatory pain (D’Souza et al. 2011; Kuyinu et al. 2016). MIA induces chondrocyte cell death and bone lesions by interfering with a key glycolytic enzyme. MIA induction in the OA model increases inflammatory cytokines and causes pain, which gradually leads to nerve cell damage, nervous irritability, and neuralgia (Kuyinu et al. 2016). In this study, we aimed to determine the effect of PEA supplementation and its molecular mechanism of action in an experimental OA animal model.

## Materials and methods

### Materials

PEA (Levagen^®^) was provided by Gencor Pacific Ltd, Hong Kong. Diclofenac sodium salt, MIA, 4',6-diamidino-2-phenylindole (DAPI), and hematoxylin and eosin (H&E) were purchased from Sigma-Aldrich Co. (St. Louis, MO, USA). Enzyme-linked immunosorbent assay (ELISA) kits for prostaglandin E2 (PGE_2_), leukotriene B4 (LTB_4_), tumor necrosis factor-α (TNF)-α, and interleukin (IL)-1β were purchased from R&D Systems (Minneapolis, MN, USA). Fluorochrome-conjugated secondary antibodies (Alexa 488 and 564) were purchased from Thermo Fisher Scientific (Waltham, MA, USA). Safranin O was purchased from ScienCell Research Laboratories (Carlsbad, CA, USA), and anti-aggrecan and anti-collagen type II alpha 1 (COL2A1) antibodies were purchased from Santa Cruz Biotechnology (Santa Cruz, CA, USA).

### Animals

All experiments using animals reported herein were performed according to protocols approved by the Institutional Animal Care and Use Committee of Hallym University (HallymR1 2018-75). Six-week-old male Sprague–Dawley (SD) rats were purchased from DooYeol Biotech (Seoul, Korea). The rats were housed under specific pathogen-free conditions with a temperature of 23 ± 3 °C, relative humidity of 50% ± 10%, 10–15 times ventilation, 150–200 lx illumination, and a 12-h light/dark cycle. The rats were fed a commercial non-purified rodent diet (Cargill Agri Purina, Seongnam, Republic of Korea) with free access to water.

### Induction of MIA-induced OA in rats

After acclimation for 1 week, MIA-induced OA rat models were generated as described previously (Udo et al. 2016). The rats were anesthetized with 2–3% isoflurane/N_2_O/O_2_ mixture vapor using an inhalation anesthesia apparatus and ventilator (Fluovac System; Harvard Apparatus, Holliston, MA, USA). After anesthetization, 3 mg of MIA in 50 μL of saline was injected into the intra-articular joint of the right knee in all rats except those in the normal control group. Rats in the control group received an injection of saline instead of the MIA solution. One day after the injection of MIA, all rats were randomly divided into five groups: (1) normal control group (injected with saline + treated with phosphate-buffered saline (PBS) termed NOR, *n* = 10); (2) control group (injected with MIA + treated with PBS, termed CON, *n* = 10), (3) 50 mg/kg body weight (BW)/day PEA-treated group (injected with MIA + treated with 50 mg of PEA/kg BW/day termed PEA50, *n* = 10); (4) 100 mg/kg BW/day PEA-treated group (injected with MIA + treated with 100 mg of PEA/kg BW/day termed PEA100, *n* = 10), and (5) positive control group (injected with MIA + treated with 6 mg of diclofenac/kg BW/day termed DiC, *n* = 10). The rats in each group were orally administered PEA or diclofenac once daily for 4 weeks. At the end of the experiment, isoflurane was used to anesthetize the animals and the thickness of both knees was measured with a digital caliper, and then blood samples were collected from the heart. The rats were sacrificed by carbon dioxide asphyxiation, and the knee joints and synovia were extracted for further analysis.

### Biochemical analyses of serum

The serum levels of total cholesterol, triglyceride, glucose, blood urea nitrogen (BUN), and activities of aspartate aminotransferase (AST) and alanine aminotransferase (ALT) were measured using a blood chemistry autoanalyzer (KoneLab 20XT; Thermo Fisher Scientific, Vantaa, Finland).

### Micro-computed tomography (micro-CT) analysis

To evaluate the changes in the knee joint microarchitecture, the femorotibial joint was scanned using a micro-CT scanner (VivaCT 80; Scanco Medical AG, Brüttisellen, Switzerland) with a source voltage of 70 keV, current of 114 μA, and isotropic resolution of 20 μm at the Chuncheon Center of the Korea Basic Science Institute. Scans were integrated into three-dimensional images, and the three-dimensional morphometric parameters were calculated using micro-CT scanner image analysis software provided with the micro-CT scanner. The bone surface/bone volume (BS/BV, %) of the subchondral bone in the femorotibial joint was analyzed to evaluate the degree of bone erosion. The bone volume fraction (bone volume/total volume, BV/TV, %), trabecular thickness (Tb.Th, mm), and trabecular number (Tb.N, 1/mm) of the metaphysis of the tibia were analyzed to evaluate structural changes caused by various treatments.

### Histological analysis

The knee joints were fixed with 4% paraformaldehyde, decalcified, embedded in paraffin, and cut into 5-μm-thick sections. The sectioned tissues were stained with safranin O and H&E. A light microscope (Axio Imager; Carl Zeiss Meditec AG, Jena, Germany) was used to observe the stained tissues. The randomly selected fields of the slides were photographed and examined in a blinded manner. The severity of knee joint OA was evaluated according to the Osteoarthritis Research Society International (OARSI) (Pritzker et al. 2006; Glasson et al. 2010).

### Measurement of nitric oxide (NO)

As an indicator of NO production in the sera, the nitrite concentration in the sera was determined using the Griess Reagent System (Promega, Madison, WI, USA) in accordance with the manufacturer’s instructions.

### ELISA

The levels of PGE_2_, LTB_4_, TNF-α, and IL-1β in sera were measured using ELISA kits according to the manufacturer’s instructions.

### Immunofluorescence (IF) staining

Paraffin-embedded knee joint tissues were sectioned to a thickness of 5 μm, deparaffinized, and blocked using 5% bovine serum albumin. The indicated antibodies and fluorochrome-conjugated secondary antibodies (Alexa 488 or 564) were used to perform IF staining. DAPI was used to counterstain the nuclei. The randomly selected fields of the slides were photographed at  × 400 magnification and examined in a blinded manner. The Axio Imager microscope and AxioVision software (Carl Zeiss) were used to quantify the immune-positive cells.

### Quantitative real-time reverse transcription-polymerase chain reaction (RT-PCR)

The total RNA was extracted from the synovia of the knee joints using TRIzol Reagent (Invitrogen Life Technologies, Carlsbad, CA, USA) according to the manufacturer’s instructions. A micro-volume UV–Vis Spectrophotometer (BioSpec-nano, Shimadzu, Kyoto, Japan) was used to determine the content and purity of the total RNA. The total RNA (2 μg) was reverse transcribed into complementary single stranded DNA using the HyperScript RT Master Mix kit (GeneAll Biotechnology, Seoul, Korea). Real-time PCR was performed using the Rotor-Gene SYBR Green PCR Kit (Qiagen, Valencia, CA, USA) and Rotor-Gene 3000 instrument (Corbett Research, Mortlake, Australia), according to the manufacturer’s instructions. Table 1 shows the nucleic acid sequences of the primers used in this study. The results were analyzed using Rotor-Gene 6000 Series software (Corbett Research, version 6). The relative expression levels of target genes were normalized to those of glyceraldehyde 3-phosphate dehydrogenase (*Gapdh*).

**Table 1:** Primer sequences used in this study

	Forward primer (5'–3')	Reverse primer (5'–3')
*iNos*	CACCACCCTCCTTGTTCAAC	CAATCCACAACTCGCTCCAA
*Cox-2*	TGCGATGCTCTT CCGAGCTGTGCT	TCAGGAAGTTCCTTATTTCCTTTC
*5-Lox*	CCATCCAGCTCAACCAAACC	GATGTGTGCGGAGAAGATGG
*Tnf-α*	AAATGGGCTCCCTCTCATCAGTTC	TCTGCTTGGTGGTTTGCTACGAC
*Il-1β*	CACCTCTCAAGCAGAGCACAG	GGGTTCCATGGTGAAGTCAAC
*Mmp-2*	TGGGGGAGATTCTCACTTTG	CCATCAGCGTTCCCATACTT
*Mmp-3*	TGGGAAGCCAGTGGAAATG	CCATGCAATGGGTAGGATGAG
*Mmp-9*	TGCTCCTGGCTCTAGGCTAC	TTGGAGGTTTTCAGGTCTCG
*Mmp-13*	TGGCGACAAAGTAGATGCTG	TGGCATGACTCTCACAATGC
*Timp-1*	CTGAGAAGGGCTACCAGAGC	GTCATCGAGACCCCAAGGTA
*Gapdh*	CTCAACTACATGGTCTACATGTTCCA	CTTCCCATTCTCAGCCTTGACT

### Statistical analysis

All data are expressed as mean ± standard error of the mean of at least three independent experiments. GraphPad Prism 5.0 (GraphPad Software, San Diego, CA, USA) was used for statistical analysis. Differences between groups were analyzed using Student’s *t* test or the one-way analysis of variance, and statistical significance was set at *P* < 0.05.

## Results

### Oral administration of PEA caused no adverse effects

Initially, we measured the BW of the rats during the experimental period and conducted a blood biochemical analysis to determine whether PEA administration caused adverse effects in vivo. Intra-articular injection of MIA or oral administration of PEA did not affect BW gain, serum levels of glucose and total cholesterol, BUN, and ALT activity. Serum triglyceride levels considerably increased with intra-articular MIA injection, and this increase noticeably reduced with the administration of PEA at 100 mg/kg BW. The administration of PEA considerably reduced AST activity, which tended to increase with intra-articular MIA injection. On the contrary, the administration of diclofenac noticeably reduced BW gain and serum glucose levels, compared with those in the NOR and CON groups (Table 2). These results indicate that there were no overt adverse effects in PEA-treated rats.

**Table 2:** Effect of PEA on body weight gain and blood biochemistry index in MIA-induced OA rats

	NOR	CON	PEA50	PEA100	DiC
Initial body weight (g)	180.9 ± 2.9	180.7 ± 2.8	180.9 ± 1.6	180.9 ± 3.2	180.7 ± 1.1
Final body weight (g)	376.3 ± 7.3	372.0 ± 4.3	365.7 ± 5.4	360.0 ± 6.3	355.7 ± 5.1^#^
Body weight gain (g)	195.4 ± 7.0	191.4 ± 5.3	184.8 ± 6.1	179.1 ± 6.4	175.0 ± 5.2^#^
Glucose (mg/dL)	168.1 ± 11.7	182.9 ± 12.6	177.9 ± 11.9	167.7 ± 5.0	142.7 ± 9.2^#^
Triglyceride (mg/dL)	62.5 ± 4.8	105.0 ± 7.2***	87.6 ± 6.6	83.7 ± 5.8^#^	92.6 ± 8.7
Total cholesterol (mg/dL)	58.3 ± 1.1	60.4 ± 2.6	57.1 ± 1.4	60.2 ± 1.4	61.1 ± 1.9
BUN (mg/dL)	11.7 ± 0.6	13.1 ± 0.4	13.9 ± 0.4	14.0 ± 0.6	13.7 ± 0.5
AST (U/L)	120.0 ± 15.3	143.9 ± 13.6	94.6 ± 4.2^##^	103.1 ± 7.3^#^	105.7 ± 6.9^#^
ALT (U/L)	38.6 ± 3.1	42.3 ± 3.7	38.8 ± 2.1	36.7 ± 1.3	38.0 ± 6.9

Values are expressed as mean ± SEM (*n* = 10) ^*^P < 0.05, ^**^P < 0.01, ^***^P < 0.001 significantly different from the NOR group. ^#^*P* < 0.05, ^##^*P* < 0.01, ^###^*P* < 0.001 significantly different from the CON group

*BUN* blood urea nitrogen, *AST* aspartate aminotransferase, *ALT* alanine aminotransferase, *PEA* palmitoylethanolamide, *OA* osteoarthritis, *MIA* monosodium iodoacetate, *NOR* normal control group (injected with saline + treated with phosphate-buffered saline (PBS)), *CON* control group (injected with MIA + treated with PBS), *PEA50* or *PEA100* 50 or 100 mg/kg body weight (BW)/day PEA-treated group (injected with MIA + treated with 50 or 100 mg of PEA/kg BW/day), *DiC* positive control group (injected with MIA + treated with 6 mg of diclofenac/kg BW/day)

### Mitigation of knee joint swelling by PEA

The thickness of the knee joint was measured to examine whether PEA administration mitigated the swelling of the knee joint. There was no observable difference in the left knee joint thickness without MIA injection in any of the experimental groups. The right knee joint thickness of the CON group was considerably higher than that of the NOR group. The administration of PEA decreased the thickness of the knee joints compared with that in the CON group. In addition, the right/left knee joint thickness ratio substantially reduced in the PEA100 group compared with that in the CON group (Table 3).

**Table 3:** Effect of PEA on knee thicknesses in MIA-induced OA rats

	NOR	CON	PEA50	PEA100	DiC
Left (normal) knee thicknesses (mm)	10.56 ± 0.14	10.60 ± 0.07	10.41 ± 0.14	10.54 ± 0.12	10.28 ± 0.03
Right (OA) knee thicknesses (mm)	10.47 ± 0.07	12.37 ± 0.11***	11.88 ± 0.24	11.92 ± 0.14^#^	11.19 ± 0.11^###^
Right knee/left knee ratio	1.00 ± 0.01	1.17 ± 0.01***	1.14 ± 0.02	1.13 ± 0.01^#^	1.09 ± 0.01^###^

Values are expressed as mean ± SEM (*n* = 10). ^*^P < 0.05, ^**^P < 0.01, ^***^P < 0.001 significantly different from the NOR group. ^#^*P* < 0.05, ^##^*P* < 0.01, ^###^*P* < 0.001 significantly different from the CON group

*PEA* palmitoylethanolamide, *OA* osteoarthritis, *MIA* monosodium iodoacetate, *NOR* normal control group (injected with saline + treated with phosphate-buffered saline (PBS)), *CON* control group (injected with MIA + treated with PBS), *PEA50* or *PEA100* 50 or 100 mg/kg body weight (BW)/day PEA-treated group (injected with MIA + treated with 50 or 100 mg of PEA/kg BW/day), *DiC* positive control group (injected with MIA + treated with 6 mg of diclofenac/kg BW/day)

### PEA ameliorated cartilage degradation

**Fig. 1:**
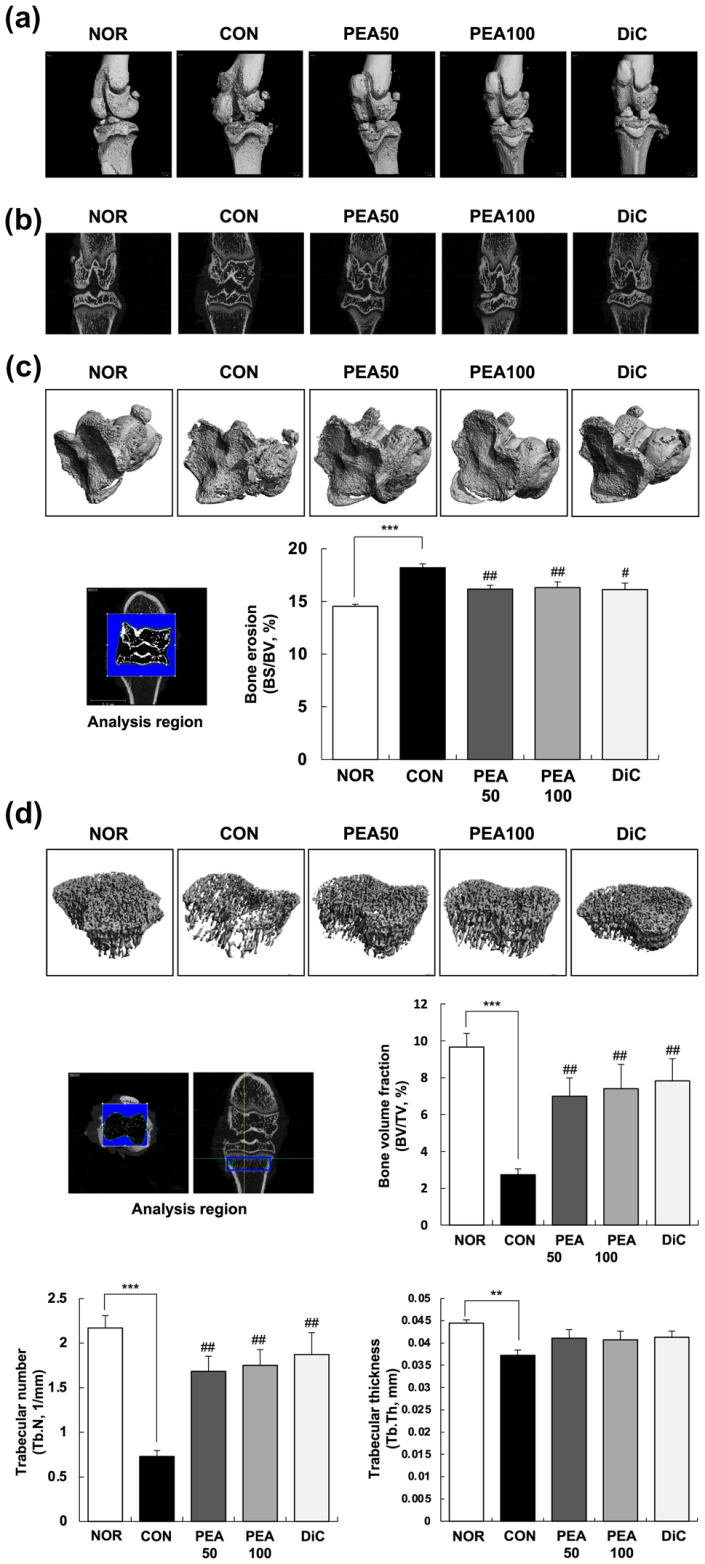
Effect of PEA on OA symptoms in MIA-induced OA rats. MIA-induced OA rats were administered either PEA (50 or 100 mg/kg BW/day) or diclofenac (6 mg/kg BW/day) for 4 weeks. The femorotibial joint was scanned, integrated into three-dimensional micro-CT images, and analyzed with a micro-CT scanner and built-in software. **a** Three-dimensional micro-CT images. **b** Two-dimensional micro-CT images. **c** Gray scale reconstructed image for quantitative analysis and BS/BV (%) of the subchondral bone in the femorotibial joint. **d** Gray scale reconstructed image for quantitative analysis, BV/TV (%), Tb.N (1/mm), and Tb.Th (mm) of the metaphysis of the tibia. Each bar represents mean ± SEM (*n* = 5). ^*^P < 0.01, ^**^P < 0.05, and ^***^P < 0.001 significantly different from the NOR group. ^#^*P* < 0.01, ^##^*P* < 0.05, and ^###^*P* < 0.001 significantly different from the CON group. *PEA* palmitoylethanolamide, *OA* osteoarthritis, *MIA* monosodium iodoacetate, *BW* body weight, *NOR* normal control group (injected with saline + treated with phosphate-buffered saline (PBS)), *CON* control group (injected with MIA + treated with PBS), *PEA50* or *PEA100* 50 or 100 mg/kg body weight (BW)/day PEA-treated group (injected with MIA + treated with 50 or 100 mg of PEA/kg BW/day), *DiC* positive control group (injected with MIA + treated with 6 mg of diclofenac/kg BW/day), *micro-CT* micro-computed tomography, *BS/BV* bone surface/bone volume, *BV/TV* bone volume/total volume, *Tb.N* trabecular number, *Tb.Th* trabecular thickness

Micro-CT scans with three-dimensional remodeling were applied to MIA-induced OA rats to validate the anti-osteoarthritic effect of PEA. Three- and two-dimensional images from micro-CT scans revealed erosion of the subchondral and irregular articular surfaces in the CON group. The formation of subchondral erosion and irregular articular surface decreased with PEA administration (Fig. 1A, B). To quantify the degree of bone erosion, the BS/BV of the subchondral bone in the femorotibial joint was analyzed. BS/BV in the CON group was substantially higher than that in the NOR group. PEA administration substantially decreased BS/BV compared with that in the CON group (Fig. 1C). Within the metaphysis, Tb.N, Tb.Th, and BV/TV considerably decreased in the CON group compared with those in the NOR group. PEA administration considerably increased BV/TV and Tb.N compared with those in the CON group, but did not affect Tb.Th (Fig. 1D). These results indicate that PEA administration ameliorated MIA-induced cartilage degradation and joint structure deformation.

**Fig. 2:**
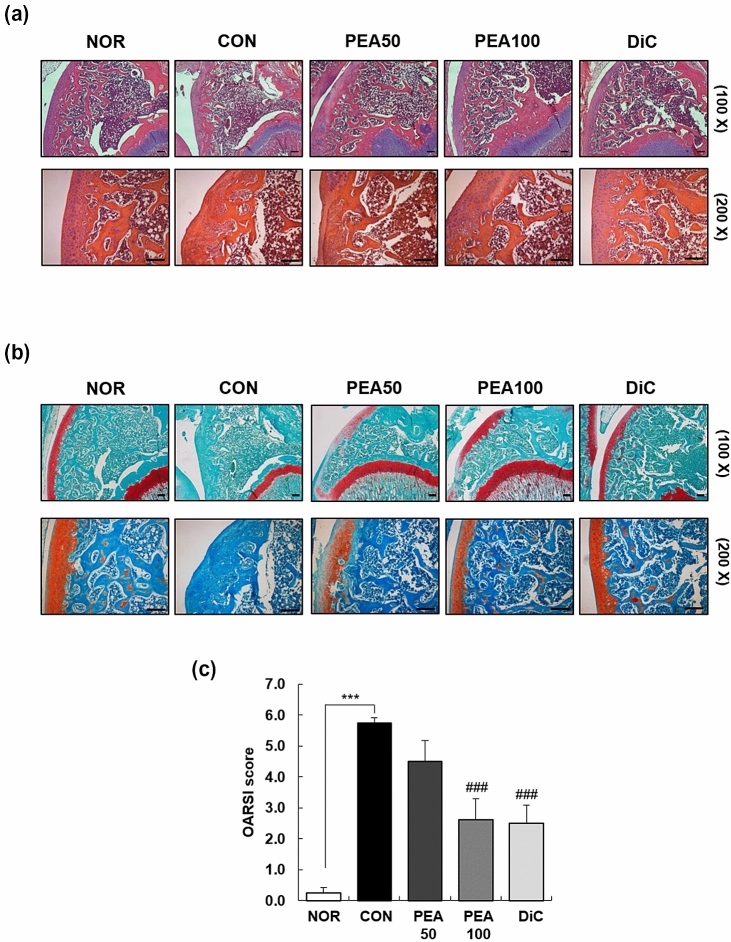
Effect of PEA on histological changes in the articular cartilage in MIA-induced OA rats. MIA-induced OA rats were administered either PEA (50 or 100 mg/kg BW/day) or diclofenac (6 mg/kg BW/day) for 4 weeks. Articular cartilage was stained with H&E (**a**) and safranin O (**b**), and subjected to Osteoarthritis Research Society International (OARSI) scoring (**c**). Representative staining images are shown. Scale bar, 50 μm. Each bar represents mean ± SEM (*n *= 5). ^*^P < 0.01, ^**^P < 0.05, and ^***^P < 0.001 significantly different from the NOR group. ^#^*P* < 0.01, ^##^*P* < 0.05, and ^###^*P* < 0.001 significantly different from the CON group. *PEA* palmitoylethanolamide, *OA* osteoarthritis, *MIA* monosodium iodoacetate, *BW* body weight, *H&E* hematoxylin and eosin, *NOR* normal control group (injected with saline + treated with phosphate-buffered saline (PBS)), *CON* control group (injected with MIA + treated with PBS), *PEA50* or *PEA100* 50 or 100 mg/kg body weight (BW)/day PEA-treated group (injected with MIA + treated with 50 or 100 mg/kg BW/day), *DiC* positive control group (injected with MIA + treated with 6 mg of diclofenc/kg BW/day)

In addition, we stained the knee cartilage with H&E and Safranin O to determine whether the administration of PEA alleviated the histopathology of OA in the knee joint. H&E staining revealed well-preserved articular cartilage in the NOR group. However, the CON group exhibited severely damaged articular cartilage, as evidenced by the abraded surface. Safranin O staining results revealed less stained areas in the CON group, which indicates degraded proteoglycan in the extracellular matrix. The PEA50, PEA100, and DiC groups showed smoother articular surfaces and higher proteoglycan volumes than the CON group (Fig. 2). These results indicate that PEA administration reversed the histopathological changes induced by intra-articular MIA injection.

### PEA mitigated MIA-induced aggrecan and COL2A1 loss in articular cartilage

**Fig. 3:**
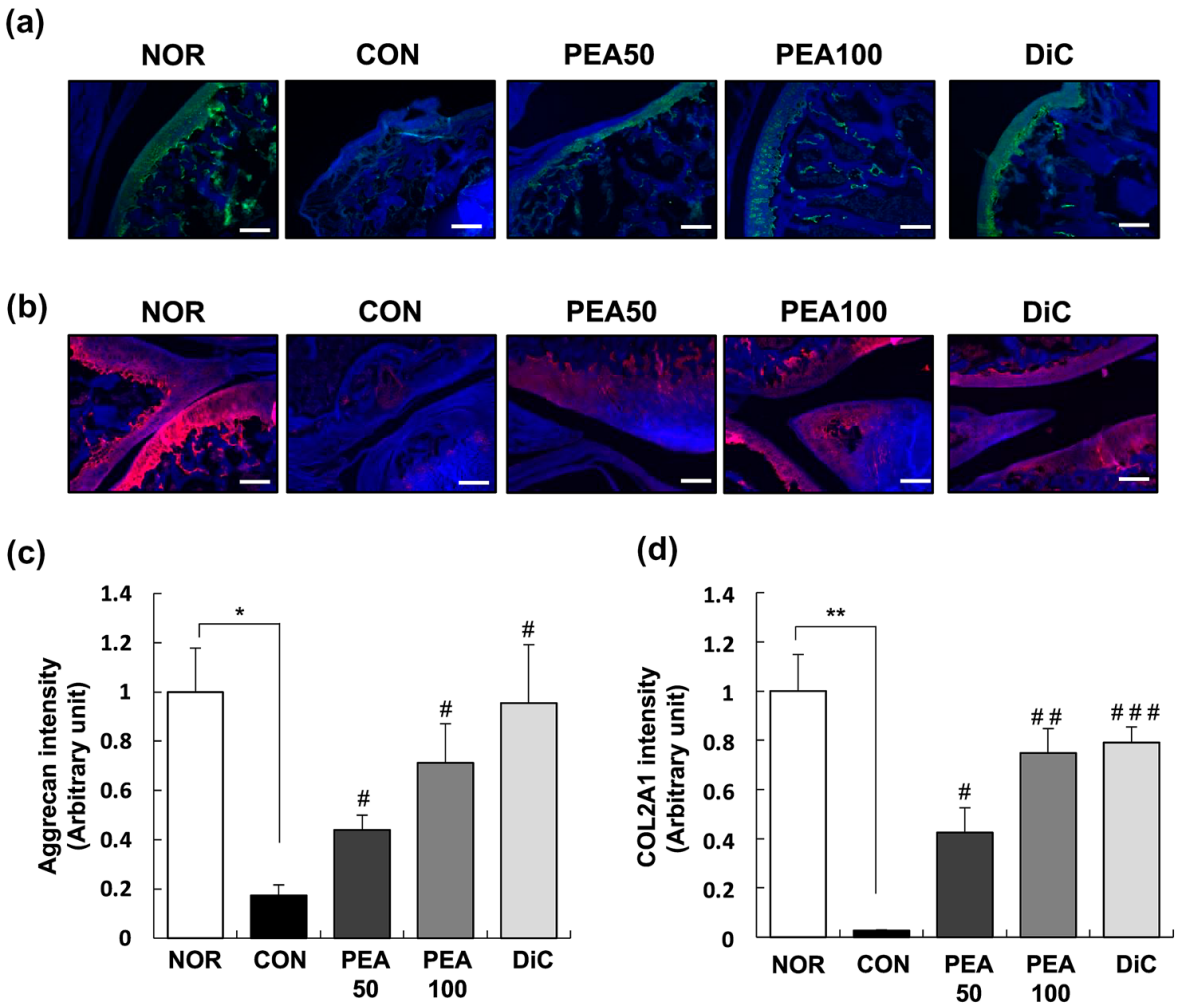
Effect of PEA on the expression of aggrecan and COL2A1 in the articular cartilage of MIA-induced OA rats. MIA-induced OA rats were administered either PEA (50 or 100 mg/kg BW/day) or diclofenac (6 mg/kg BW/day) for 4 weeks. Articular cartilage was stained with aggrecan (**a**) and COL2A1 (**b**) antibodies. Representative IF staining images are shown. Scale bar, 50 μm. (**c**), (**d**) Staining intensity of the indicated proteins was quantified. Each bar represents mean ± SEM (*n* = 5). ^*^P < 0.01, ^**^P < 0.05, and ^***^P < 0.001 significantly different from the NOR group. ^#^*P* < 0.01, ^##^*P* < 0.05, and ^###^*P* < 0.001 significantly different from the CON group. *COL2A1* collagen type II alpha 1, *PEA* palmitoylethanolamide, *IF* immunofluorescence, *OA* osteoarthritis, *MIA* monosodium iodoacetate, *BW* body weight, *NOR* normal control group (injected with saline + treated with phosphate-buffered saline (PBS)), *CON* control group (injected with MIA + treated with PBS), *PEA50* or *PEA100* 50 or 100 mg/kg body weight (BW)/day PEA-treated group (injected with MIA + treated with 50 or 100 mg of PEA/kg BW/day), *DiC* positive control group (injected with MIA + treated with 6 mg of diclofenac/kg BW/day)

IF staining results revealed that aggrecan expression considerably diminished in the articular cartilage of rats with OA induced by intra-articular MIA injection. The PEA50, PEA100, and DiC groups presented higher aggrecan expression than the CON group (Fig. 3A). Similar to aggrecan expression in the articular cartilage, COL2A1 expression considerably decreased in the CON group compared with that in the NOR group. PEA administration substantially increased COL2A1 expression reduced by intra-articular MIA injection (Fig. 3B). The expression of aggrecan and COL2A1 increased 4.1- and 27.7-fold in the PEA100 group, respectively, compared with that in the CON group (Fig. 3C, D).

### PEA alleviated inflammation in MIA-induced OA rats

The serum levels of inflammatory mediators and cytokines were measured to investigate the effects of PEA on inflammation in MIA-induced OA rats. The ELISA results showed that the levels of NO, PGE_2_, LTB_4_, TNF-α, and IL-1β considerably increased in the sera of MIA-induced OA rats, and these changes substantially reduced with PEA administration. The serum levels of NO, PGE_2_, LTB_4_, TNF-α, and IL-1β reduced by 57.8%, 14.7%, 43.7%, 22.7%, and 21.8% in the PEA100 group, respectively, compared with those in the CON group (Table 4).

**Table 4:** Effect of PEA on serum levels of inflammatory mediators and cytokines in MIA-induced OA rats

	NOR	CON	PEA50	PEA100	DiC
NO (μM)	5.75 ± 0.48	9.81 ± 1.47*	4.33 ± 0.29^##^	4.14 ± 0.26^##^	4.55 ± 0.24^##^
PGE_2_ (ng/mL)	6.43 ± 0.25	7.33 ± 0.18*	7.06 ± 0.18	6.25 ± 0.32^#^	5.13 ± 0.54^##^
LTB_4_ (ng/mL)	1.34 ± 0.08	2.08 ± 0.24*	1.26 ± 0.14^#^	1.17 ± 0.08^##^	1.23 ± 0.14^##^
TNF-α (pg/mL)	26.66 ± 0.59	32.67 ± 1.73**	25.64 ± 0.49^##^	25.24 ± 0.65^##^	26.76 ± 0.63^##^
IL-1β (pg/mL)	72.90 ± 3.02	83.88 ± 3.36*	69.41 ± 4.61^#^	65.63 ± 1.87^###^	72.60 ± 1.88^#^

Values are expressed as mean ± SEM (*n* = 10). ^*^P < 0.05, ^**^P < 0.01, ^***^P < 0.001 significantly different from the NOR group. ^#^*P* < 0.05, ^##^*P* < 0.01, ^###^*P* < 0.001 significantly different from the CON group

*OA* osteoarthritis, *PEA* palmitoylethanolamide, *MIA* monosodium iodoacetate, *NO* nitric oxide, *PGE*_*2*_ prostaglandin E2, *LTB*_*4*_ leukotriene B4, *TNF-α* tumor necrosis factor-α, *IL-1β* interleukin-1β, *NOR* normal control group (injected with saline + treated with phosphate-buffered saline (PBS)), *CON* control group (injected with MIA + treated with PBS), *PEA50* or *PEA100* 50 or 100 mg/kg body weight (BW)/day PEA-treated group (injected with MIA + treated with 50 or 100 mg of PEA/kg BW/day), *DiC* positive control group (injected with MIA + treated with 6 mg of diclofenac/kg BW/day)

**Fig. 4:**
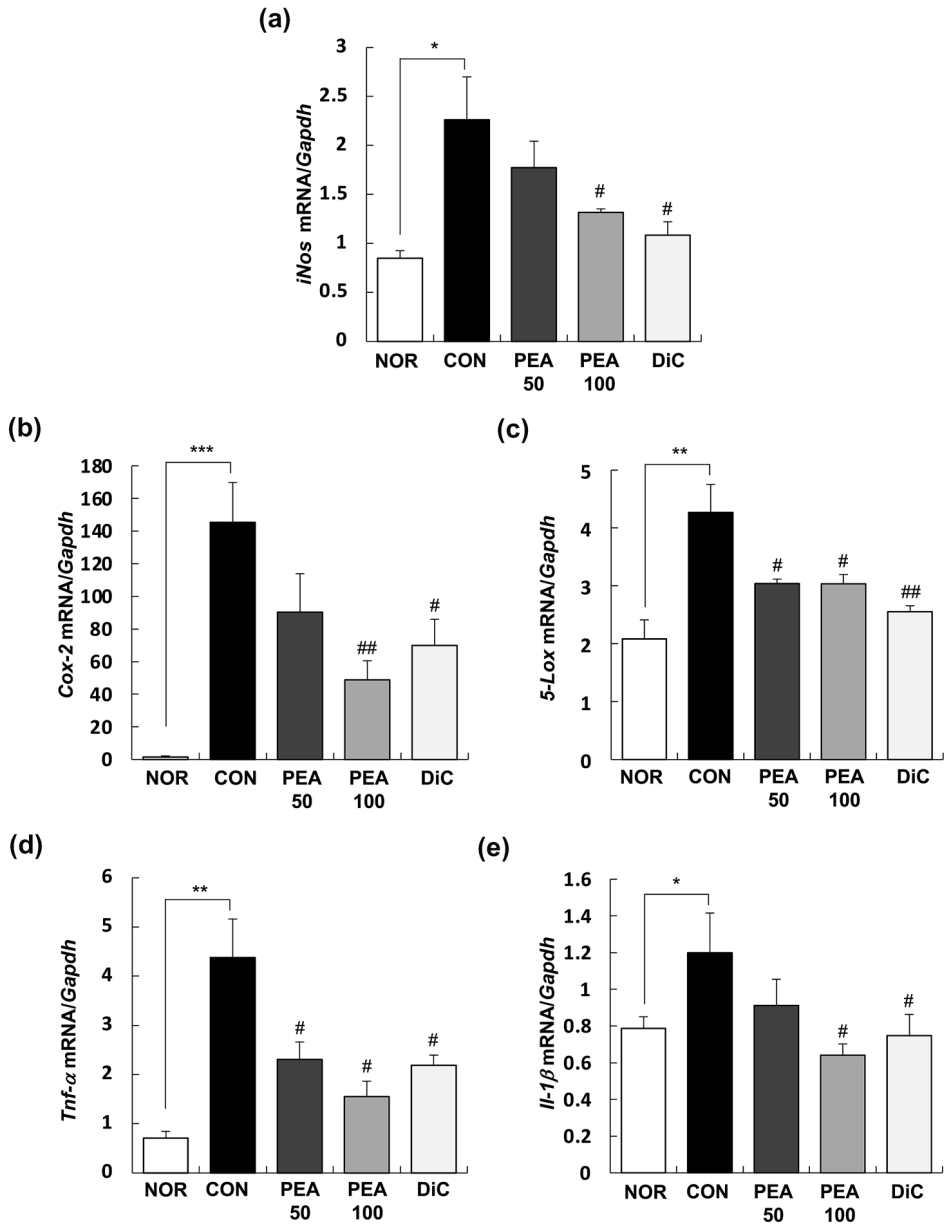
Effect of PEA on the expression of inflammatory mediators and cytokines in the synovia of MIA-induced OA rats. MIA-induced OA rats were administered either PEA (50 or 100 mg/kg BW/day) or diclofenac (6 mg/kg BW/day) for 4 weeks. The total RNA was extracted from the synovia and reverse transcribed, followed by amplification using real-time PCR. Expression of target mRNA was normalized to that of *GAPDH* and represented relative to that of the NOR group. Each bar represents mean ± SEM (*n* = 10). ^*^P < 0.01, ^**^P < 0.05, and ^***^P < 0.001 significantly different from the NOR group. ^#^*P* < 0.01, ^##^*P* < 0.05, and ^###^*P* < 0.001 significantly different from the CON group. *PEA* palmitoylethanolamide, *OA* osteoarthritis, *MIA* monosodium iodoacetate, *iNos *inducible nitric oxide synthase, *Cox-2* cyclooxygenese-2, *5-Lox* 5-lipoxygenase, *Tnf*-*α* tumor necrosis factor-α, *Il*-*1β* interleukin-1β,*Gapdh* glyceraldehyde 3-phosphate dehydrogenase, *NOR* normal control group (injected with saline + treated with phosphate-buffered saline (PBS)), *CON* control group (injected with MIA + treated with PBS), *PEA50* or *PEA100* 50 or 100 mg/kg body weight (BW)/day PEA-treated group (injected with MIA + treated with 50 or 100 mg of PEA/kg BW/day), *DiC* positive control group (injected with MIA + treated with 6 mg of diclofenac/kg BW/day)

Next, we performed real-time RT-PCR to investigate the effects of PEA on the expression of inflammatory mediators and cytokines in the synovia of the knee joints. As shown in Fig. 4, the mRNA expression of *iNos*, *5-*lipoxygenase (*Lox*), cyclooxygenase (*Cox*)*-2*, *Tnf-α*, and *Il-1β* substantially increased in the synovia of MIA-induced OA rats, which was suppressed by the administration of PEA. In the PEA100 group, the mRNA expression of *iNos*, *5-Lox*, *Cox-2*, *Tnf-α*, and *Il-1β* reduced by 41.8%, 66.4%, 28.9%, 64.6%, and 53.8%, respectively, compared with that in the CON group (Fig. 4).

### PEA modulated the expression of *Mmp* and *Timp* in the synovia of MIA-induced OA rats

**Fig. 5:**
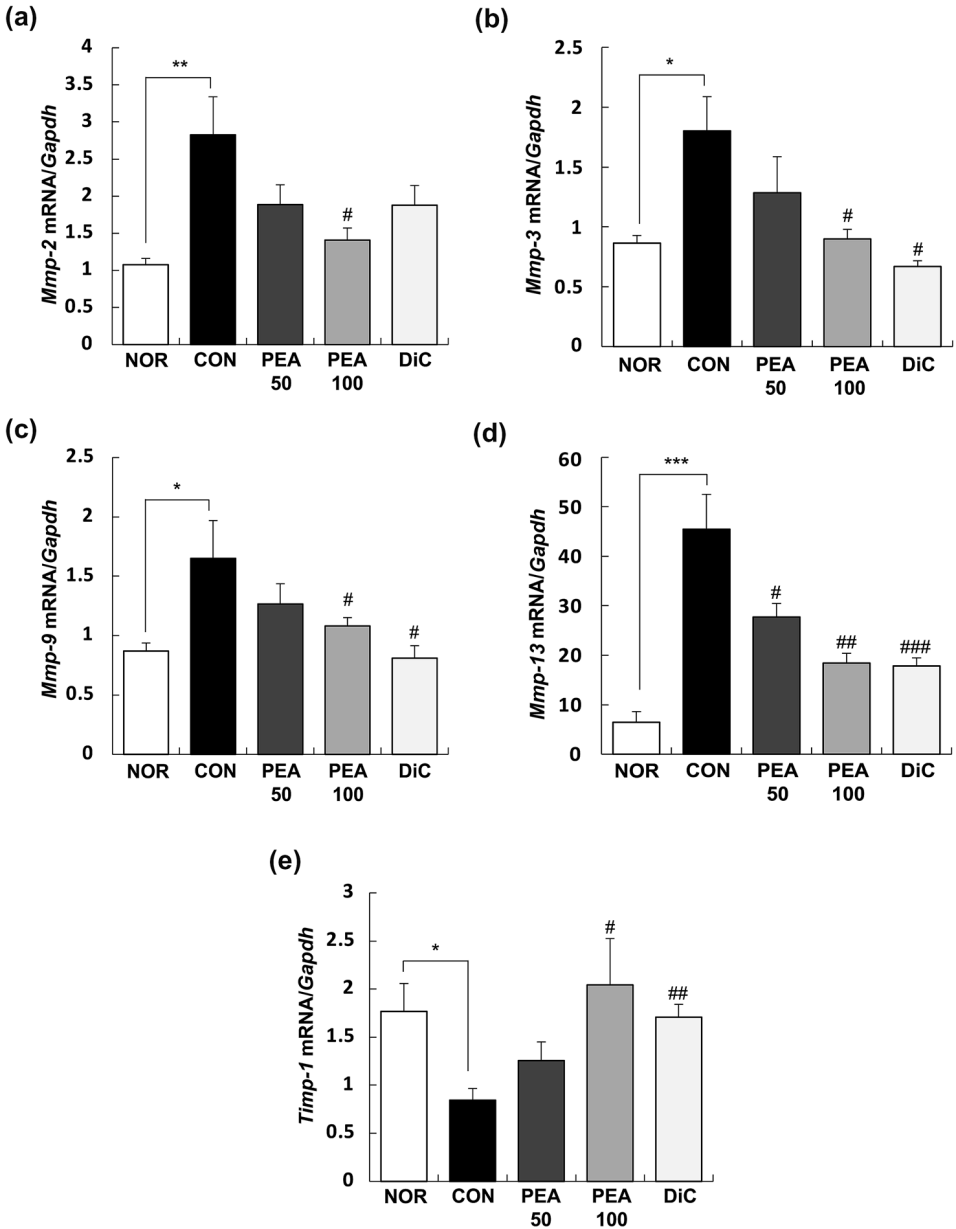
Effect of PEA on the expression of MMPs and TIMP-1 in the synovia of MIA-induced OA rats. MIA-induced OA rats were administered either PEA (50 or 100 mg/kg BW/day) or diclofenac (6 mg/kg BW/day) for 4 weeks. The total RNA was extracted from the synovia and reverse transcribed, followed by amplification using real-time PCR. Expression of target mRNA was normalized to that of *Gapdh* and represented relative to that of the NOR group. Each bar represents mean ± SEM (*n* = 10). ^*^P < 0.01, ^**^P < 0.05, and ^***^P < 0.001 significantly different from the NOR group. ^#^*P* < 0.01, ^##^*P* < 0.05, and ^###^*P* < 0.001 significantly different from the CON group. *PEA* palmitoylethanolamide, *OA* osteoarthritis, *MIA* monosodium iodoacetate, *MMP* matrix metalloproteinase, *TIMP-1* tissue inhibitor of metalloproteinase-1, *NOR* normal control group (injected with saline + treated with phosphate-buffered saline (PBS)), *CON* control group (injected with MIA + treated with PBS), *PEA50* or *PEA100* 50 or 100 mg/kg body weight (BW)/day PEA-treated group (injected with MIA + treated with 50 or 100 mg of PEA/kg BW/day), *DiC* positive control group (injected with MIA + treated with 6 mg of diclofenac/kg BW/day)

In the synovia of the knee joints, the mRNA expression of matrix metalloproteinase (*Mmp*)*-2*, *Mmp-3*, *Mmp-9*, and *Mmp-13* noticeably increased with intra-articular MIA injection, and these changes substantially reduced with PEA administration at 100 mg/kg BW. Meanwhile, tissue inhibitor of metalloproteinase (*Timp*)*-1* mRNA expression considerably reduced in the CON group, but this reduction was prevented by PEA administration at 100 mg/kg BW (Fig. 5).

## Discussion

We confirmed that PEA mitigated the symptoms of MIA-induced knee OA based on the following findings: (1) PEA reduced swelling of the knee joint (Table 3), (2) PEA ameliorated cartilage degradation and joint structure deformation (Fig. 1), (3) PEA reversed histopathological changes (Fig. 2), and (4) PEA mitigated aggrecan and COL2A1 loss in the articular cartilage (Fig. 3). PEA did not cause any adverse effects. PEA treatment alleviated hypertriglyceridemia and increased AST levels in MIA-injected rats (Table 2). Another common adverse effect of arthritis medication is hypomotility (Alsalem et al. 2019). In our study, MIA-induced OA rats that were administered diclofenac, an arthritis drug, had substantially reduced physical activity throughout the day and did not respond to external stimuli. However, when PEA was administered, the physical activity of the rats considerably recovered (data not shown).

The main symptoms of OA are joint cartilage degeneration, chronic pain, and synovial inflammation (Saklatvala 1986). In our study, the right knees of rats that were administered MIA showed cartilage degradation and decreased bone volume fraction, trabecular number and thickness, and articular cartilage damage compared with the NOR group (Fig. 1 and 2). It is necessary to maintain a healthy synovial microenvironment to maintain cartilage integrity and homeostasis. The anti-hyperanalgesic effects of PEA involve inflammatory mediators, pro-inflammatory cytokines, neutrophil infiltration, and pro-inflammatory enzymes (i.e. COX-2 and iNOS), pro-inflammatory kinases (mitogen-activated protein kinase) (Costa et al. 2008), and neurotrophic factors (i.e. nerve growth factor), mast cell degranulation through autacoid local injury antagonism, histamine, and inhibition of PGE_2_ secretion (Costa et al. 2008; Alhouayek and Muccioli 2014).

The expression of these pro-inflammatory cytokines is higher in the joint tissue of patients with OA than in healthy subjects (Richardson et al. 2008). In our study, inflammatory mediators such as NO, PGE_2_, and LTB_4_, and pro-inflammatory cytokines such as TNF-α and IL-1β considerably increased in the serum of the MIA-induced OA rats, but decreased to the same levels as those in the NOR group with PEA treatment (Table 4). In addition, the expression of *iNos*, *5-Lox*, *Cox-2*, *Tnf-α*, and *Il-1β *increased in the synovia of the knee joints with MIA treatment, but considerably decreased with PEA treatment (Fig. 4).

Inflammation occurs in joints via inflammatory mediators and pro-inflammatory cytokines, which in turn stimulate the secretion of cartilage-degrading enzymes such as MMP, thereby increasing cartilage degradation (Chadjichristos et al. 2003). We analyzed the mRNA expression of *Mmp-2*, *Mmp-3*, *Mmp-9*, *Mmp-13*, and *Timp-1* in the synovia of the knee joints to determine whether PEA directly affects *Mmp* expression. The mRNA expression of all four types of MMPs increased substantially and that of TIMP-1 decreased with intra-articular MIA injection. These changes decreased in a dose-dependent manner and recovered to a similar degree as that of the DiC group when 100 mg/kg BW PEA was administered (Fig. 5). In an experimental animal model of MIA-induced OA, PEA improved motor function and protected cartilage (Britti et al. 2017). PEA can relieve knee OA symptoms and protect the joints in patients with mild OA (Steels et al. 2019).

PEA is a cannabinoid-like compound, and the endocannabinoid system has emerged as a popular target for the development of new pain relievers (La Porta et al. 2014). Structurally, NAEs and PEA act via unique mechanisms related to several biological pathways (Impellizzeri et al. 2013). The direct molecular targets of PEA are PPAR-α and GPR55, and the indirect molecular targets are transient receptor potential vanilloid type-1 channel and cannabinoid-1 and -2 receptors (Petrosino and Di Marzo 2017; Guida et al. 2017). PEA directly or indirectly activates these molecular targets or receptors to exert its unique actions. In other words, when PEA activates PPAR-α, PPAR-α reduces the expression of anti-inflammatory proteins such as IκB-α, which interferes with the translocation of nuclear factor (NF)-κB and inhibits the expression of the pro-inflammatory protein TNF-α. Ultimately, the recruitment of immune cells is reduced (Alhouayek and Muccioli 2014). Regarding OA, PPAR-α is involved in inhibiting inflammation and preventing the development of chondrogenic factors (Fahmi et al. 2001). PPAR-α decreases the expression of MMP-9 and TGF-β (Wang et al. 2016). WY-14643, a potent PPAR-α agonist, inhibits the expression of metalloproteinases such as MMP-1, -3, and -13 in cartilage explants of human OA (Clockaerts et al. 2011). WY-14643 also exhibits anti-inflammatory effects by inhibiting pro-inflammatory factors such as NO, PGE_2_, MCP-1, IL-6, IL-1β, and TNF-α through the NF-κB pathway (Huang et al. 2016). The anti-inflammatory effect of the PPAR-α receptor eventually leads to the inhibition of cartilage degradation. OA causes chronic pain because of the continuous inflammatory response and breakdown of joint cartilage, thus controlling the inflammatory response and cartilage degeneration is crucial for treatment (Alsalem et al. 2019).

In summary, PEA alleviates cartilage degradation and knee joint swelling in MIA-induced OA rats via a decrease in inflammatory mediators, such as NO, PGE_2_, LTB_4_, TNF-α, and IL-1β, and suppression of the secretion of cartilage-degrading enzymes, such as MMP-1, MMP-3, MMP-9, and MMP-13. Thus, PEA may be a potential novel therapeutic drug for OA.

## Data Availability

The dataset generated during the present study is available upon reasonable request to the corresponding author (Eun Ji Kim).
